# Identification of lysosome-related genes in connection with prognosis and immune cell infiltration for drug candidates in head and neck cancer

**DOI:** 10.1515/biol-2022-0660

**Published:** 2023-08-09

**Authors:** Ye Shen, Haibin Chen, Xiaoyang Gong, Ziyi Wang, Mengjie Chen, Donghui Chen

**Affiliations:** Department of General Surgery, Affiliated Aoyang Hospital of Jiangsu University, Zhangjiagang, 215600, China; Department of Otorhinolaryngology & Head and Neck Surgery, The First Affiliated Hospital of Nanjing Medical University, Nanjing, 210029, China; Aoyang Cancer Institute, Affiliated Aoyang Hospital of Jiangsu University, Zhangjiagang, 215600, China; Department of Otolaryngology Head & Neck Surgery, Changhai Hospital of Navy Medical University, Shanghai 200433, China

**Keywords:** head and neck squamous cell carcinoma, molecular docking, immune cell infiltration, lysosome

## Abstract

Lysosome dysfunction has been shown to play an important role in cancer progression. However, few research studies have reported the role of lysosomes in head and neck squamous cell carcinoma (HNSCC) progression. Lysosome-related genes (LRGs) were collected from the Molecular Signatures Database. Differentially expressed lysosome-related genes (DELRGs) were identified from the TCGA-HNSCC dataset. The least absolute shrinkage and selection operator and multivariate Cox regression analysis were used to identify the prognostic genes. The prognostic values and expression of hub DELRGs were further validated by GEO datasets. Estimation of STromal and Immune cells in MAlignant Tumors using Expression data and the single-sample gene set enrichment analysis were applied to evaluate the correlation between cathepsin G (CTSG) and immune infiltrates. Twenty-two DELRGs were identified. Among them, CTSG was an independent prognostic biomarker for HNSCC patients. Gene set enrichment analysis indicated that the potential mechanism of CTSG in regulating HNSCC was associated with the immune- and inflammation-related pathways. CTSG expression was highly correlated with immune cell infiltration. Finally, two potential compounds (CH and MAN) targeting CTSG protein were identified, and their reliability was validated through molecular docking analysis. CTSG was associated with immune infiltration and had prognostic value in HNSCC patients, which may be a potential biomarker for predicting the outcome of immunotherapy.

## Graphical Abstract

**Figure:**
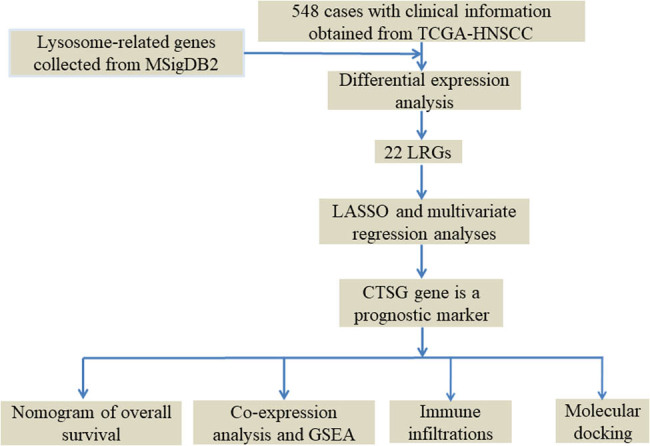

## 1 Introduction

Head and neck cancer is the world’s sixth leading cause of cancer [1]. It accounts for 7% of all cancers, with approximately 344,000 deaths and 693,000 new cases reported worldwide each year [1]. The majority of head and neck cancers arise in the larynx, throat, and mucous membrane epithelium of the mouth and are known as head and neck squamous cell carcinoma (HNSC) [2]. Despite the considerable advances made in the treatment of malignancies, there has been no significant improvement in head and neck cancer mortality rates in recent decades [3]. In addition, the 5-year survival rate for those diagnosed with HNSC is only about 50%. Immunotherapy has gained much attention in recent years; however, it is only effective in between 20 and 30% of patients [4]. In view of the increase in incidence and the low survival rates of HNSC, and to better tailor diagnostic, preventive, and therapeutic approaches for HNSC, there is an urgent need to develop biomarkers that can accurately predict tumor prognosis [5,6].

Lysosomes play a vital role in cellular aging, development and homeostasis as signaling hubs, and degradation centers within cells [7]. Moreover, complex transcriptional programs regulated lysosomal composition and biogenesis, tailoring their activity to changing cellular needs [8]. It is not surprising that alterations in these fundamental activities are critical to the pathophysiology of diseases, including neurodegenerative disorders, pancreatitis, autoimmune disorders, atherosclerosis, and cancer [9,10,11,12,13]. Thus, unraveling lysosome-related genes (LRGs) will help us to understand the underlying pathological mechanisms and thereby develop new therapeutic approaches relevant to human diseases. Furthermore, lysosomal dysfunction is associated with cancer-associated vascularization, tumor spread, immune escape, cancer cell metastasis, invasion, energy metabolism, and proliferation [14]. Therefore, knowing what influences lysosomes and how they might be regulated will help in cancer diagnosis and prognosis prediction. However, limited studies have reported the role of genes involved in lysosome functions in tumorigenesis and progression.

In the present study, we systematically analyzed LRGs in HNSC. The least absolute shrinkage and selection operator (LASSO) and multivariate Cox regression analyses were used to identify the potential prognostic genes for HNSC patients. Furthermore, a series of bioinformatics approaches were used to systematically analyze the prognosis and potential functions of LRGs in HNSC patients.

## 2 Materials and methods

### 2.1 Collection of microarray data

The transcriptome and relevant clinical data of HNSC patients (44 normal samples and 504 tumor samples) were obtained from the TCGA dataset. The gene expression profile and survival data from the GSE6631 (22 normal samples and 22 tumor samples) and GSE41613 (97 tumor samples) datasets were downloaded from the GEO database. Lysosome-related data sets were collected from the Molecular Signatures Database. After removing duplicates, 163 LRGs were obtained.

### 2.2 Identification of the differentially expressed lysosome-related genes (DELRGs) in HNSC patients

DELRGs were filtered between the normal and tumor groups using the “limma” package of R. The conditions for filtering were set to false discovery rate (FDR) <0.05 and |log FC|> 1. The results were visualized using the “ggplot2” package.

### 2.3 Screening of potential prognostic biomarkers in HNSC patients

The “glmnet” and “survival” packages were used to perform the LASSO analysis based on the above DELRGs. Finally, multivariate Cox regression analysis was performed to further screen signature genes. The relationship between DELRGs and overall survival was assessed and survival curves were plotted using the “ggplot2” and “survminer” packages.

### 2.4 Construction of nomogram

Based on the results of multivariate analysis, a nomogram was established to predict overall survival in HNSC patients. Nomogram was generated and calibration curves were plotted using the “ggplot2” and “survival” R packages.

### 2.5 Identification of differentially expressed genes (DEGs) and functional enrichment analysis between the low- and high-cathepsin G (CTSG) HNSC subgroups

Based on the median CTSG expression value, we divided HNSC patients into low- and high-CTSG subgroups. DEGs were identified between the two subgroups using the “limma” package of R. The conditions for the filtering were set to FDR <0.05 and |log FC|> 1. The results were visualized using the “ggplot2” package. To assess the potential biological functions and signaling pathways of DEGs, gene set enrichment analysis (GSEA) was performed using the “clusterProfiler” package of R. The “ggplot2” package was used to visualize enrichment analysis results.

### 2.6 Immune infiltration analysis

The Estimation of STromal and Immune cells in MAlignant Tumors using Expression data (ESTIMATE) Score, Immune Score, and Stromal Score were compared between the low- and high-CTSG subgroups using the ESTIMATE software. In addition, we used the single-sample gene set enrichment analysis (ssGSEA) to compare the number of 24 immune cell types between the two subgroups. The “ggplot2” package was used to perform the correlation analysis and visualize the results. Tumor Immune Estimation Resource (TIMER) is an online analysis tool for comprehensive analysis of tumor-infiltrating immune cells (https://cistrome.shinyapps.io/timer/) [15]. The TISCH (http://tisch.comp-genomics.org) database is a comprehensive collection of single-cell RNA sequencing data that provide insights into the heterogeneity of the tumor microenvironment across different datasets and cell types [16]. We used this database to explore the heterogeneity of the tumor microenvironment in various cells.

### 2.7 Molecular docking

First, we screened the candidate drugs targeting CTSG genes by using the Dgibd database (https://dgidb.org/) [17]. The 3D protein structure of CTSG was downloaded from the PDB database (www.rcsb.org). The selection of suitable target proteins was based on the following criteria: (1) the crystal resolution was below 3.5 Å, (2) they must be obtained from *Homo sapiens*, and (3) the structure with the best resolution. Solvent and organics were removed from the protein conformations using PyMOL software. Subsequently, we performed molecular docking analysis using AutoDock software (1.5.6), and molecular docking results were visualized using PyMOL software.

## 3 Results

### 3.1 Identification of DELRGs in HNSC

**Figure 1:**
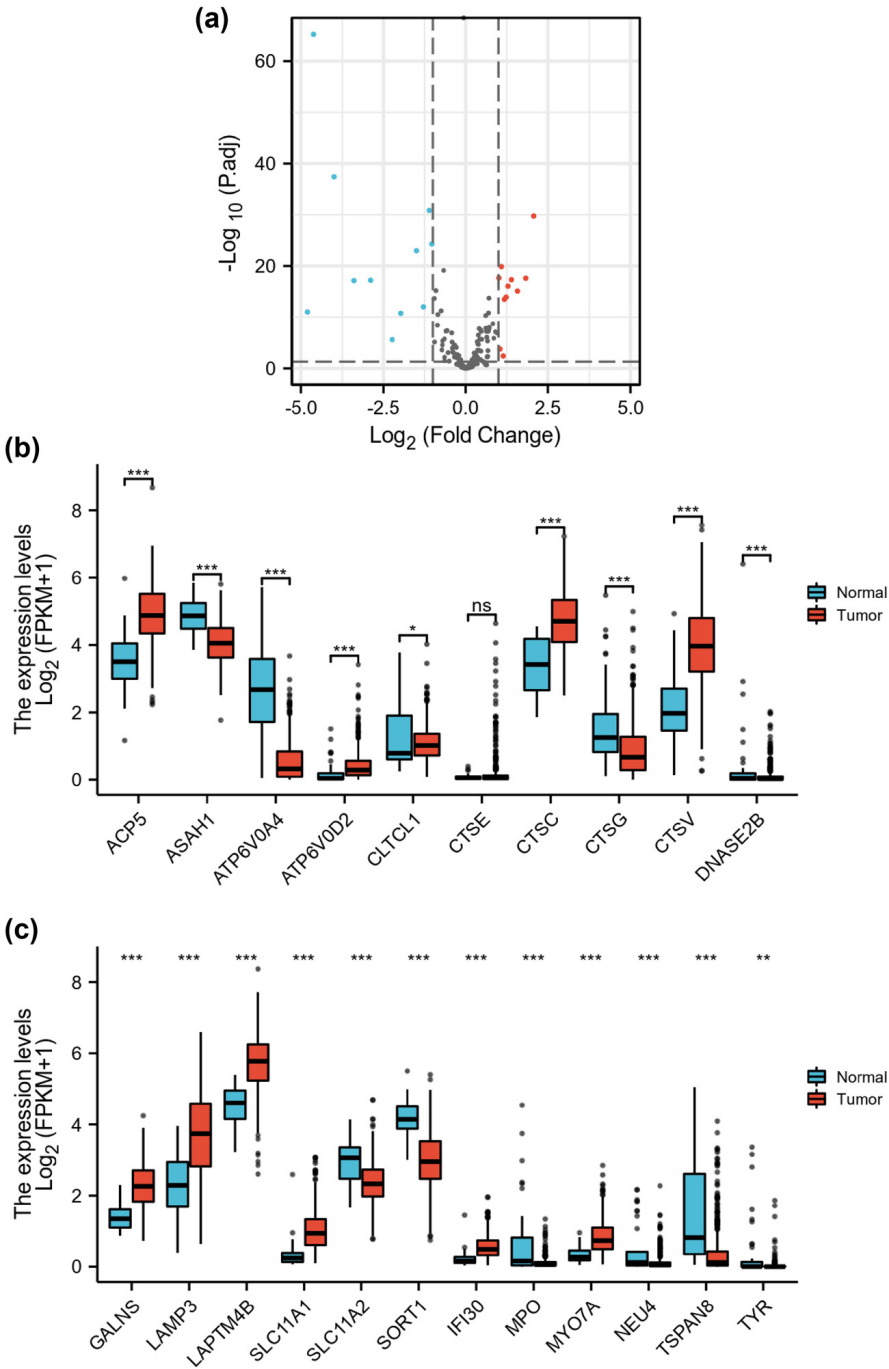
Identification of DELRGs in HNSC. (a) The volcano plot presents the LRGs. Blue dots indicate downregulated genes and red dots indicate upregulated genes. (b and c) The bar charts present the DELRG expression levels between the normal and tumor groups. *p < 0.05, **p < 0.01, ***p < 0.001, and ns indicates no significant difference.

DELRGs were presented in the volcano plot (Figure 1a). There were 11 downregulated LRGs and 11 upregulated LRGs between the normal and tumor groups. As shown in Figure 1b and c, the mRNA expression of ACP5, ATP6V0D2, CLTCL1, CTSC, CTSV, GALNS, LAMP3, LAPTM4B, SLC11A1, IFI30, and MYO7A was significantly upregulated in HNSC patients, whereas the mRNA expression of ASAH1, ATP6V0A4, CTSG, DNASE2B, SLC11A2, SORT1, MPO, NEU4, TSPAN8, and TYP was significantly downregulated in HNSC patients (*p* < 0.05).

### 3.2 Screening of potential prognostic biomarkers in HNSC patients

**Figure 2:**
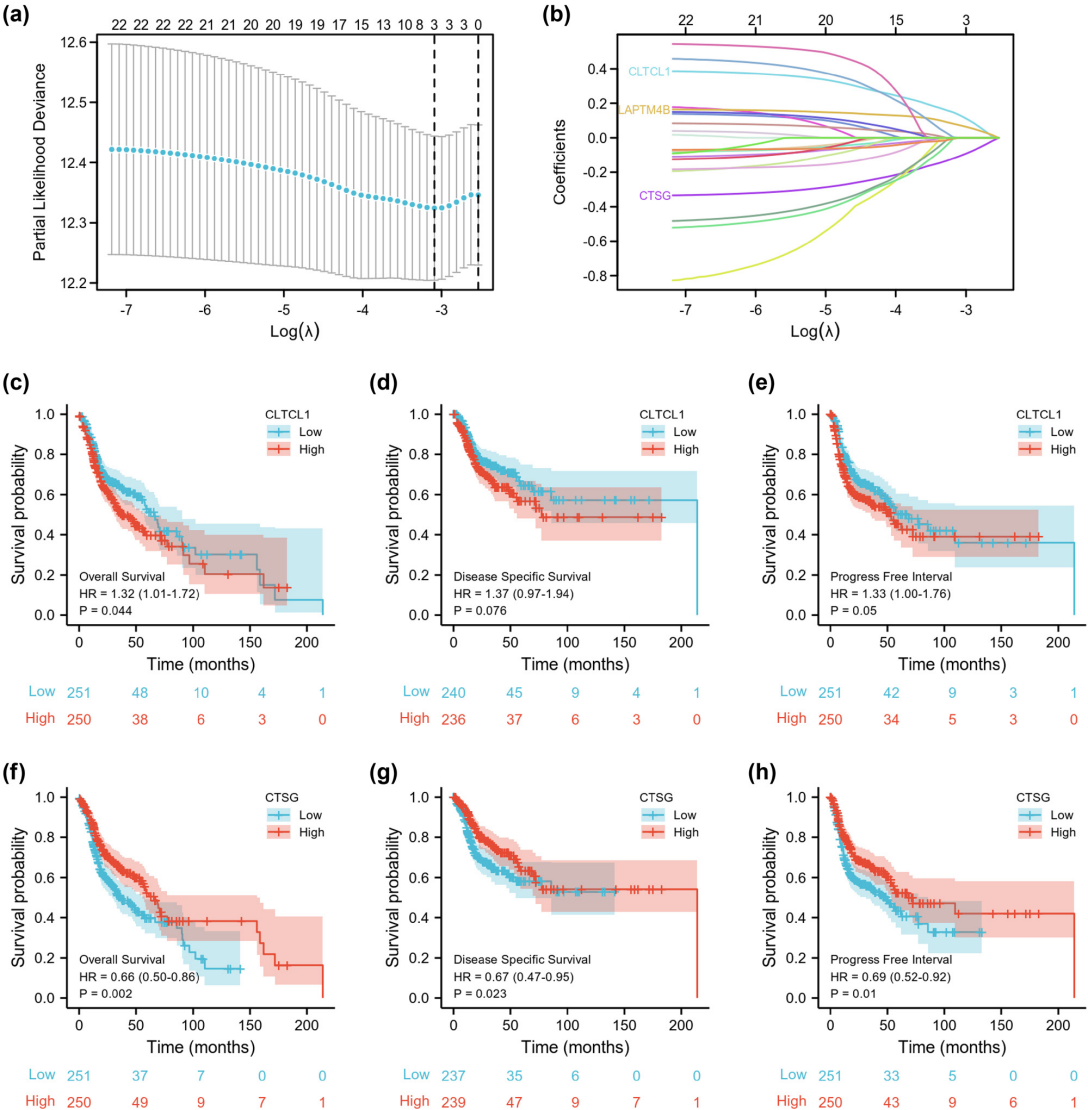
Screening of potential prognostic biomarkers for HNSC patients. (a and b) LASSO analysis identifies the prognostic biomarkers in the TCGA dataset. Effect of CLTCL1 genes on overall survival (c), disease-specific survival (d), and progress-free interval (e). Effect of CTSG genes on overall survival (f), disease-specific survival (g), and progress-free interval (h).

In the present study, the above 22 DELRGs were used to perform the LASSO regression analysis. Three DELRGs (LAPTM4B, CTSG, and CLTCL1) with non-zero coefficients were screened in HNSC patients (Figure 2a and b). Subsequently, a multivariate regression analysis was performed on these three DELRGs. The results showed that M stage, CTSG, and CLTCL1 were the independent prognostic factors in HNSC patients (Table 1).

**Table 1:** Results of univariate and multivariate regression analyses

Characteristics	Total (*N*)	Univariate analysis		Multivariate analysis	
		Hazard ratio (95% CI)	*p* value	Hazard ratio (95% CI)	*p* value
T stage	486				
T1	33	Reference			
T2	143	1.086 (0.568–2.074)	0.803		
T3	131	1.461 (0.769–2.773)	0.247		
T4	179	1.249 (0.665–2.344)	0.490		
N stage	479				
N0	238	Reference			
N1	80	1.058 (0.728–1.539)	0.768	1.020 (0.696–1.494)	0.919
N2&N3	161	1.404 (1.038–1.900)	**0.028**	1.369 (1.005–1.865)	**0.047**
M stage	476				
M0	471	Reference			
M1	5	4.745 (1.748–12.883)	**0.002**	3.945 (1.434–10.852)	**0.008**
LAPTM4B	501				
Low	251	Reference			
High	250	1.161 (0.888–1.518)	0.275		
CTSG	501				
Low	251	Reference			
High	250	0.656 (0.499–0.861)	**0.002**	0.651 (0.493–0.860)	**0.002**
CLTCL1	501				
Low	251	Reference			
High	250	1.316 (1.007–1.720)	**0.044**	1.389 (1.055–1.830)	**0.019**

Bold values represents a significant difference.

In addition, we performed a comprehensive survival analysis of prognostic genes. As shown in Figure 2c–h, only CLTCL1 genes could predict overall survival (*p* = 0.04), while CTSG genes could predict overall survival (*p* = 0.002), disease-specific survival (*p* = 0.023), and progress-free interval (*p* = 0.01).

### 3.3 Validation of expression and prognostic values of CLTCL1 and CTSG

**Figure 3:**
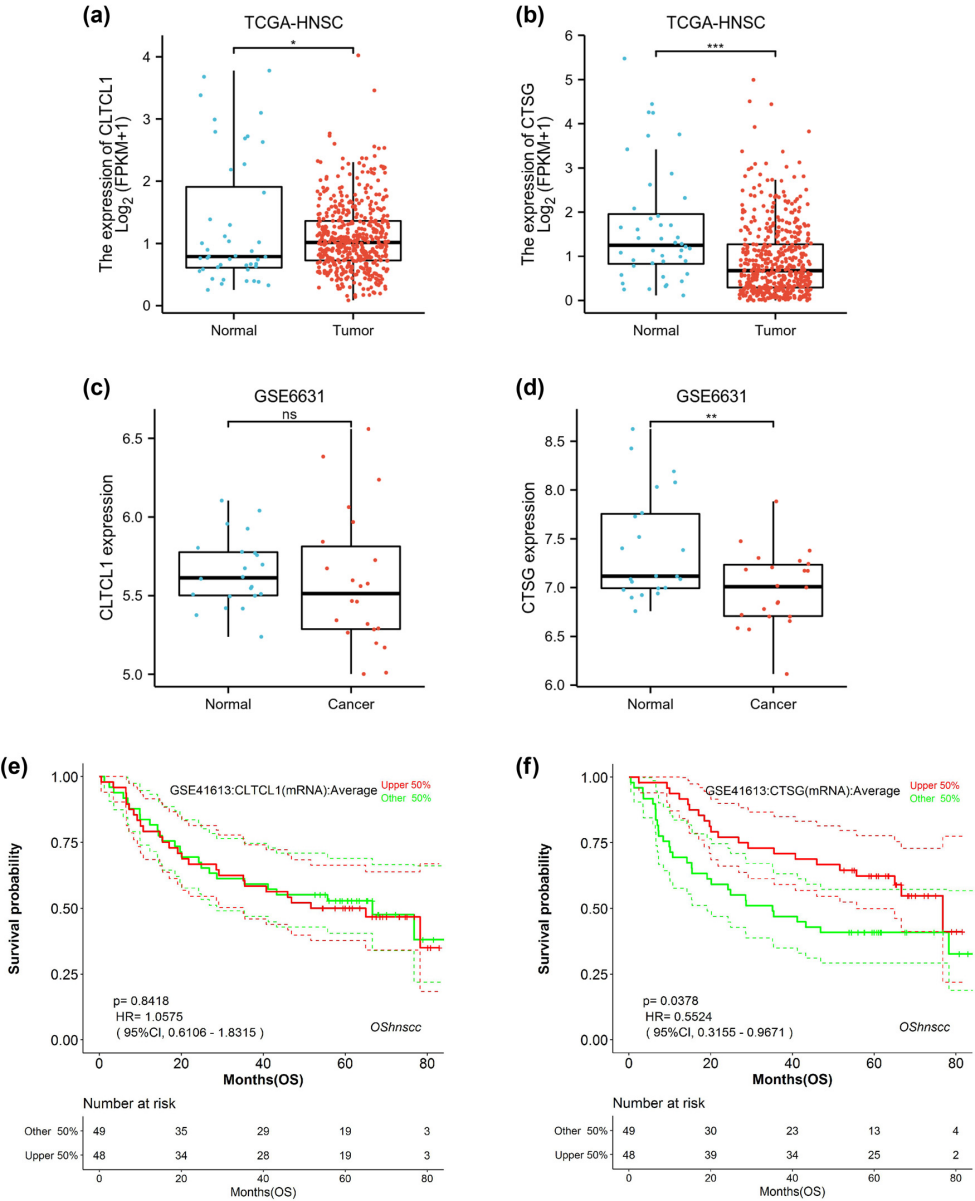
Validation of expression and prognostic values of CLTCL1 and CTSG. The scatter plots present the CLTCL1 (a) and CTSG (b) expression levels between the normal and tumor groups in the TCGA database. The scatter plots present the CLTCL1 (c) and CTSG (d) expression levels between the normal and tumor groups in the GSE6631 database.*p < 0.05, **p < 0.01, ***p < 0.001, and ns indicates no significant difference. Effect of CLTCL1 (e) and CTSG (f) genes on overall survival in the GSE41613 dataset.

As shown in Figure 3a and b, the gene expression of CLTCL1 and CTSG was significantly downregulated in HNSC patients in the TCGA-HNSC dataset. However, in the GSE6631 dataset, CLTCL1 expression was not significantly different between normal and tumor groups (Figure 3c). CTSG expression was significantly downregulated in the cancer group (*p* < 0.01, Figure 3d). We also validated the prognostic values of CLTCL1 and CTSG in the GSE41613 dataset. As shown in Figure 3e and f, CLTCL1 genes could not predict overall survival (*p* = 0.84), while CTSG genes could predict overall survival in HNSC patients (*p* = 0.0378). These findings further demonstrated that low expression of CTSG was associated with poor prognosis in HNSC patients.

As shown in Table 2, downregulation of CTSG in HNSC was significantly associated with the primary therapy outcome (*p* = 0.003), race (*p* = 0.044), anatomic neoplasm subdivision (*p* = 0.005), and overall survival (*p* = 0.015). In addition, as shown in Figure S1, the downregulation of CTSG was associated with a poor prognosis in the subgroup of CR therapy outcome (*p* = 0.005), radiation therapy (*p* = 0.008), males (*p* = 0.021), M0 stage (*p* = 0.02), N0 stage (*p* = 0.007), T2 stage (*p* = 0.041), lymph-node neck dissection (*p* = 0.002), smokers (*p* = 0.001), and age >60 (*p* = 0.002).

**Table 2:** Relationship of CTSG expression with clinicopathological features in HNSC

Characteristics	Low expression of CTSG	High expression of CTSG	*p*
*n*	251	251	
T stage, *n* (%)			0.110
T1	12 (2.5%)	21 (4.3%)	
T2	69 (14.2%)	75 (15.4%)	
T3	60 (12.3%)	71 (14.6%)	
T4	100 (20.5%)	79 (16.2%)	
N stage, *n* (%)			0.396
N0	116 (24.2%)	123 (25.6%)	
N1	34 (7.1%)	46 (9.6%)	
N2	83 (17.3%)	71 (14.8%)	
N3	3 (0.6%)	4 (0.8%)	
M stage, *n* (%)			0.682
M0	232 (48.6%)	240 (50.3%)	
M1	3 (0.6%)	2 (0.4%)	
Clinical stage, *n* (%)			0.268
Stage I	6 (1.2%)	13 (2.7%)	
Stage II	44 (9%)	51 (10.5%)	
Stage III	49 (10%)	53 (10.9%)	
Stage IV	143 (29.3%)	129 (26.4%)	
Radiation therapy, *n* (%)			0.061
No	68 (15.4%)	86 (19.5%)	
Yes	155 (35.1%)	132 (29.9%)	
Primary therapy outcome, *n* (%)			**0.003**
PD	29 (6.9%)	12 (2.9%)	
SD	1 (0.2%)	5 (1.2%)	
PR	5 (1.2%)	1 (0.2%)	
CR	176 (42.1%)	189 (45.2%)	
Gender, *n* (%)			0.190
Female	60 (12%)	74 (14.7%)	
Male	191 (38%)	177 (35.3%)	
Race, *n* (%)			**0.044**
Asian	3 (0.6%)	7 (1.4%)	
Black or African-American	31 (6.4%)	16 (3.3%)	
White	210 (43.3%)	218 (44.9%)	
Age, *n* (%)			1.000
≤60	123 (24.6%)	122 (24.4%)	
>60	128 (25.5%)	128 (25.5%)	
Histologic grade, *n* (%)			0.300
G1	33 (6.8%)	29 (6%)	
G2	139 (28.8%)	161 (33.3%)	
G3	66 (13.7%)	53 (11%)	
G4	1 (0.2%)	1 (0.2%)	
Anatomic neoplasm subdivision, *n* (%)			**0.005**
Alveolar ridge	12 (2.4%)	6 (1.2%)	
Base of tongue	10 (2%)	13 (2.6%)	
Buccal mucosa	12 (2.4%)	10 (2%)	
Floor of mouth	39 (7.8%)	22 (4.4%)	
Hard palate	2 (0.4%)	5 (1%)	
Hypopharynx	8 (1.6%)	2 (0.4%)	
Larynx	53 (10.6%)	58 (11.6%)	
Lip	0 (0%)	3 (0.6%)	
Oral cavity	32 (6.4%)	40 (8%)	
Oral tongue	52 (10.4%)	74 (14.7%)	
Oropharynx	8 (1.6%)	1 (0.2%)	
Tonsil	23 (4.6%)	17 (3.4%)	
Smoker, *n* (%)			1.000
No	56 (11.4%)	55 (11.2%)	
Yes	190 (38.6%)	191 (38.8%)	
Alcohol history, *n* (%)			0.406
No	83 (16.9%)	75 (15.3%)	
Yes	160 (32.6%)	173 (35.2%)	
Lymphovascular invasion, *n* (%)			0.379
No	108 (31.7%)	111 (32.6%)	
Yes	67 (19.6%)	55 (16.1%)	
Lymphnode neck dissection, *n* (%)			0.924
No	46 (9.2%)	44 (8.8%)	
Yes	204 (40.9%)	205 (41.1%)	
OS event, *n* (%)			**0.015**
Alive	128 (25.5%)	156 (31.1%)	
Dead	123 (24.5%)	95 (18.9%)	
Age, median (IQR)	61 (53, 69)	61 (54, 68.75)	0.789

Bold values represents a significant difference.

### 3.4 Pan-cancer analysis of CTSG expression

As shown in Figure S2, we found that CTSG was significantly downregulated in a variety of cancers, such as bladder urothelial carcinoma, breast invasive carcinoma, cervical squamous cell carcinoma and endocervical adenocarcinoma, cholangio carcinoma, colon adenocarcinoma, esophageal carcinoma, HNSC, kidney chromophobe, liver hepatocellular carcinoma, lung adenocarcinoma, lung squamous cell carcinoma, prostate adenocarcinoma, rectum adenocarcinoma, stomach adenocarcinoma, and endometrial carcinoma. CTSG was significantly upregulated in thyroid carcinoma.

### 3.5 Construction of a nomogram for HNSC patients

**Figure 4:**
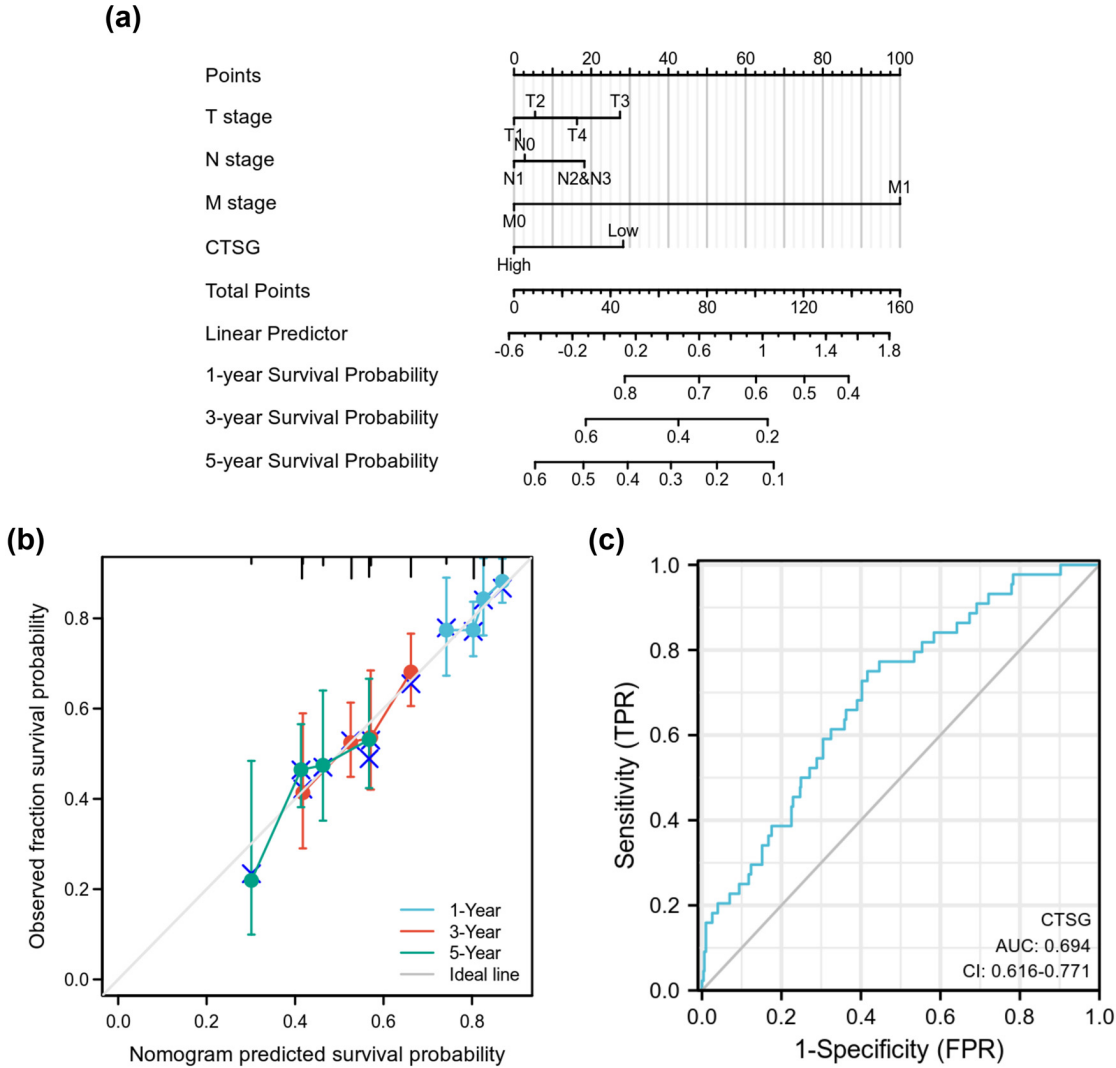
Establishment of a nomogram for HNSC patients. (a) A nomogram for evaluating the survival probability of 1-, 3-, and 5 years for HNSC. (b) Calibration curve of the nomogram. (c) ROC curve of CTSG.

As shown in Figure 4a, we constructed a nomogram and it could predict 1-, 3-, and 5-year overall survival of HNSC patients. In addition, the prediction result of the nomogram was highly consistent with the observation of HNSC patients, based on the nomogram calibration curve (Figure 4b). The diagnostic value of CTSG was assessed by the receiver operating characteristic (ROC) curve, and the area under the curve of CTSG was 0.694 (Figure 4c).

### 3.6 Screening of CTSG-associated DEGs in HNSC patients

**Figure 5:**
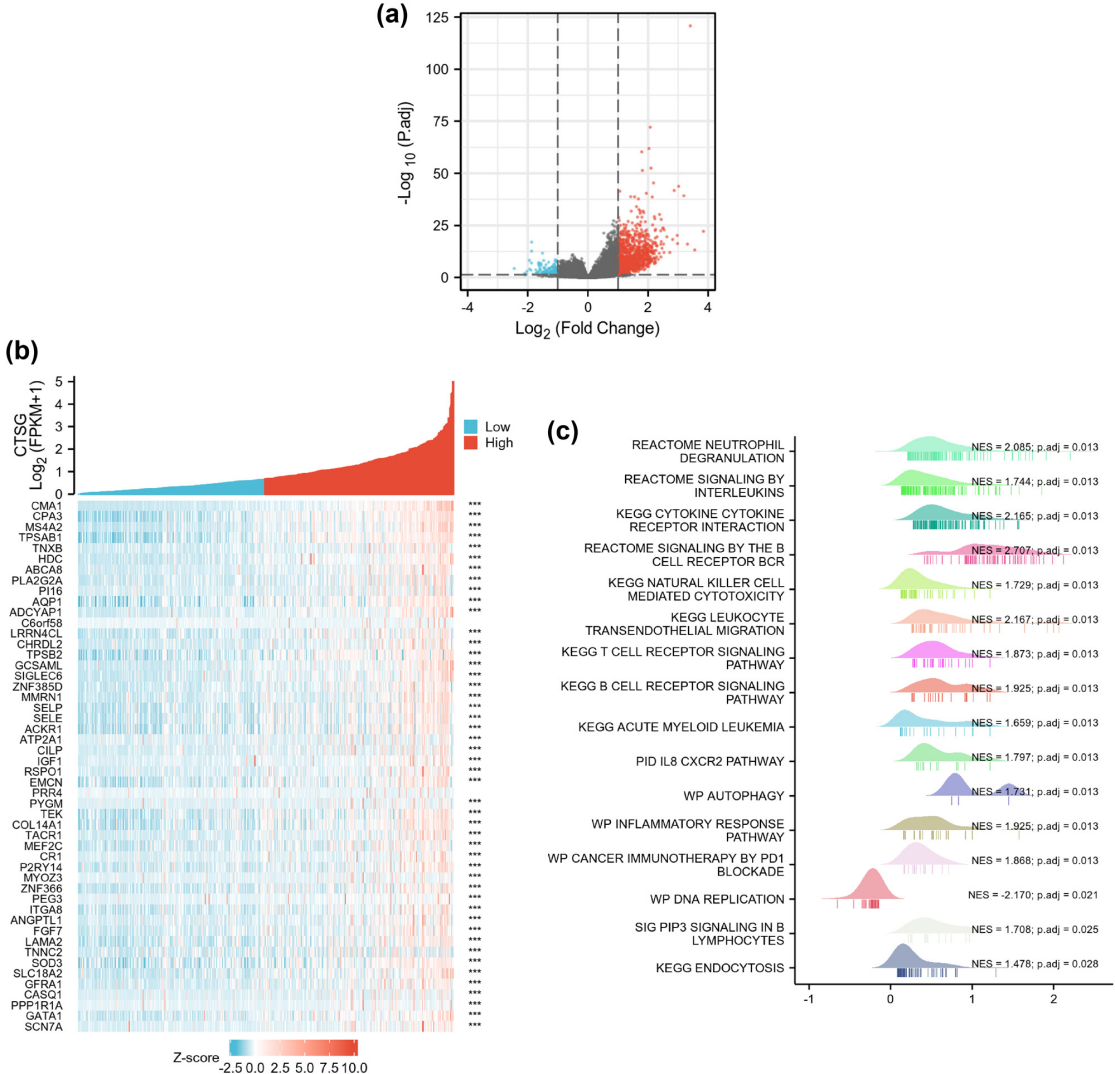
Screening of CTSG-associated DEGs in HNSC. (a) The volcano plot presents the DEGs. Blue dots indicate down-regulated genes and red dots indicate up-regulated genes. (b) Co-expression heatmap of top 50 DEGs. (c) Enrichment analysis of DEGs between the low- and high-CTSG subgroups.

As shown in Figure 5a, a total of 641 CTSG-related DEGs were identified, which included 62 downregulated genes and 579 upregulated genes. The heatmap showed the expression of the top 50 DEGs (Figure 5b). Furthermore, the GSEA was performed to further explore the function of DEGs associated with CTSG expression in HNSC patients. CTSG-related DEGs were involved in immune-related pathways, including neutrophil degranulation (NES = 2.08, *p*-adj < 0.05), signaling by interleukins (NES = 1.74, *p*-adj < 0.05), cytokine–cytokine receptor interaction (NES = 2.16, *p*-adj < 0.05), signaling by B cell receptor BCR (NES = 2.7, *p*-adj < 0.05), natural killer cell-mediated cytotoxicity (NES = 1.72, *p*.adj < 0.05), leukocyte transendothelial migration (NES = 2.16, *p*-adj < 0.05), T-cell receptor signaling pathway (NES = 1.87, *p*-adj < 0.05), B-cell receptor signaling pathway (NES = 1.92, *p*-adj < 0.05), acute myeloid leukemia (NES = 1.65, *p*-adj < 0.05), inflammatory response pathway (NES = 1.92, *p*-adj < 0.05), cancer immunotherapy by PD1 blockade (NES = 1.86, *p*-adj < 0.05), etc. (Figure 5c).

### 3.7 Correlation between the CTSG expression and the infiltration of immune cells

**Figure 6:**
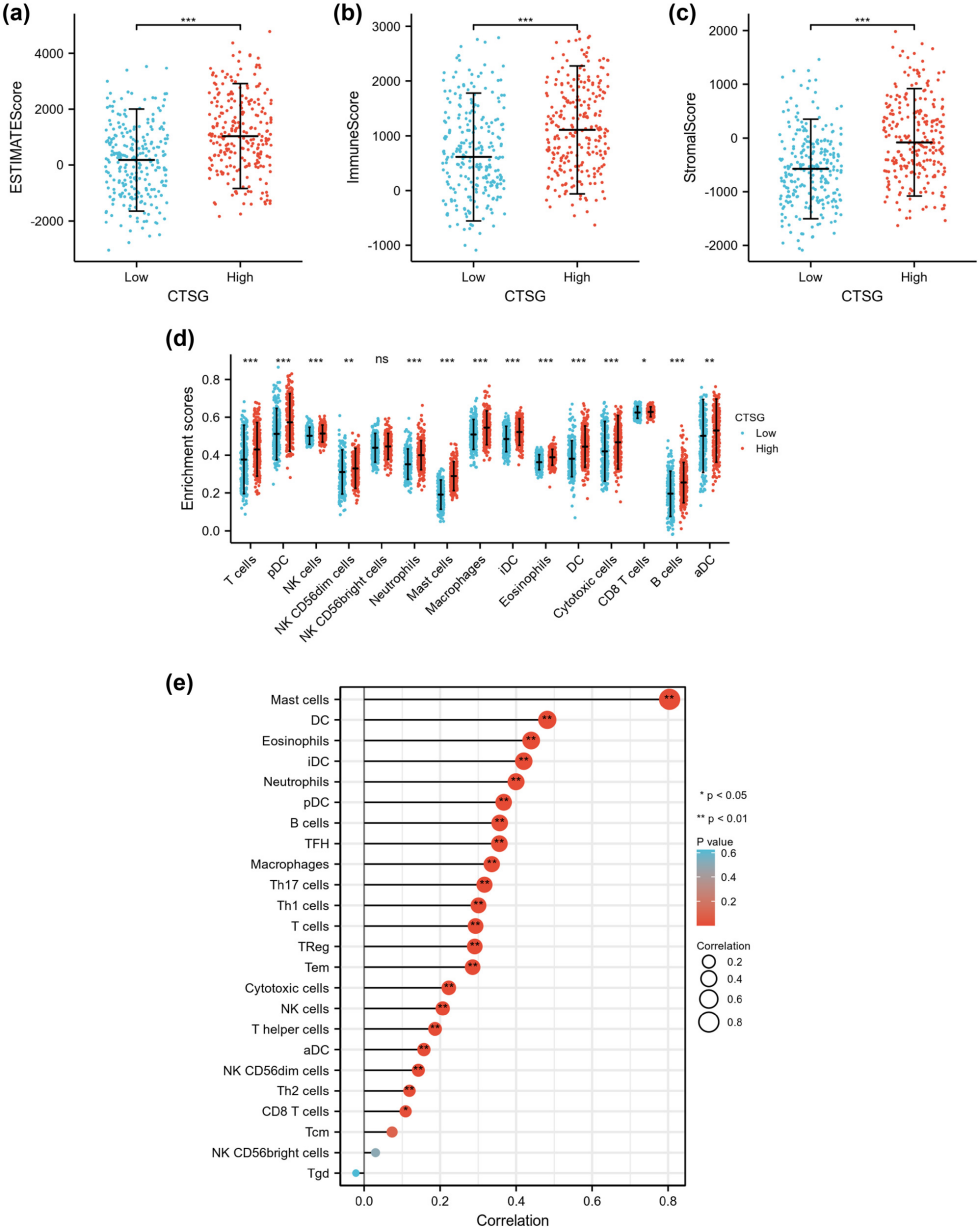
Correlation between the expression of CTSG and the infiltration of immune cells. ESTIMATE algorithm shows the relationship between CTSG expression and ESTIMATE score (a), immune score (b), and Stromal score (c). (d) ssGSEA algorithm showed the relationship between CTSG expression and immune cell infiltration. (e) The correlation results between CTSG genes and immune cell infiltration were visualized by a lollipop plot. *p < 0.05, **p < 0.01, and ***p < 0.001.

The ESTIMATE algorithm results showed that the low-CTSG subgroup had a lower ESTIMATE score (Figure 6a, *p* < 0.001), immune score (Figure 6b, *p* < 0.001), and Sromal score (Figure 6c, *p* < 0.001) compared to those in the high-CTSG subgroup. In addition, the ssGESA algorithm results indicated that the proportion of dendritic cells (DCs) (*p* < 0.001), aDC (*p* < 0.01), B cells (*p* < 0.001), CD8 T cells (*p* < 0.05), cytotoxic cells (*p* < 0.001), eosinophils (*p* < 0.001), iDC (*p* < 0.001), macrophages (*p* < 0.001), mast cells (*p* < 0.001), neutrophils (*p* < 0.001), NK CD56dim cells (*p* < 0.01), NK cells (*p* < 0.001), pDC (*p* < 0.001), and T cells (*p* < 0.001) was significantly decreased in the low-CTSG subgroup compared with those in the high-CTSG subgroup (Figure 6d). The correlation analysis revealed positive correlations between CTSG expression and most immune cells, including mast cells, DC, eosinophils, pDC, B cells, TFH, macrophages, Th17 cells, Th1 cells, T cells, TReg, Tem, cytotoxic cells, NK cells, T helper cells, aDC, NK CD56dim cells, Th2 cells, and CD8 T cells (Figure 6e).

**Figure 7:**
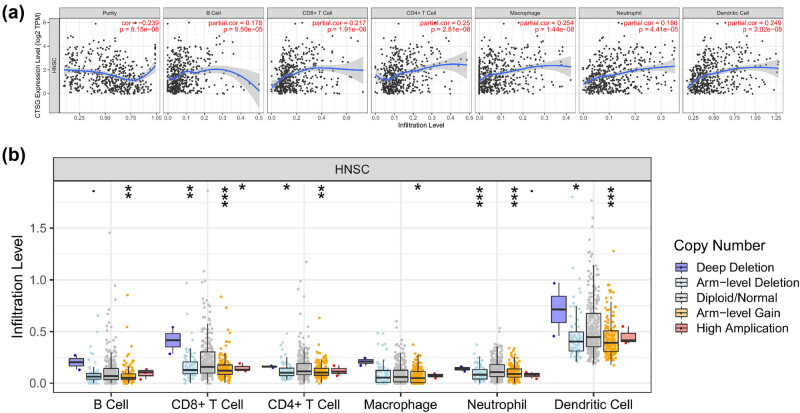
TIMER analysis tool was used to assess the relationship between CTSG and immune cell infiltration. (a) The correlation results between CTSG genes and the proportion of DC, neutrophils, macrophage, CD4+ T cells, CD8+ T cells, and tumor purity. (b) The relationship between somatic copy number alterations of CTSG and immune cell infiltration. *p < 0.05, **p < 0.01, and ***p < 0.001.

In addition, we used the TIMER analysis tool to further validate the correlation between CTSG expression and immune cell infiltration. Consistent with the above results, the expression of CTSG was positively correlated with DC (*p* = 3.02 × 10^−8^), neutrophil (*p* = 4.41 × 10^−5^), macrophage (*p* = 1.44 × 10^−8^), CD4+ T cell (*p* = 2.81 × 10^−8^), CD8+ T cell (1.91 × 10^−6^), and B cell (*p* = 9.5 × 10^−5^) (Figure 7a). Furthermore, as shown in Figure 7b, the results of copy number alterations in CTSG expression could inhibit immune cell infiltration. We analyzed the level of CTSG genes in tumor immune microenvironment-associated cells, utilizing the TISCH database. Our findings revealed that CTSG showed a higher degree of infiltration in mast cells (Figure S3).

### 3.8 Screening of potential drugs and molecular docking

**Figure 8:**
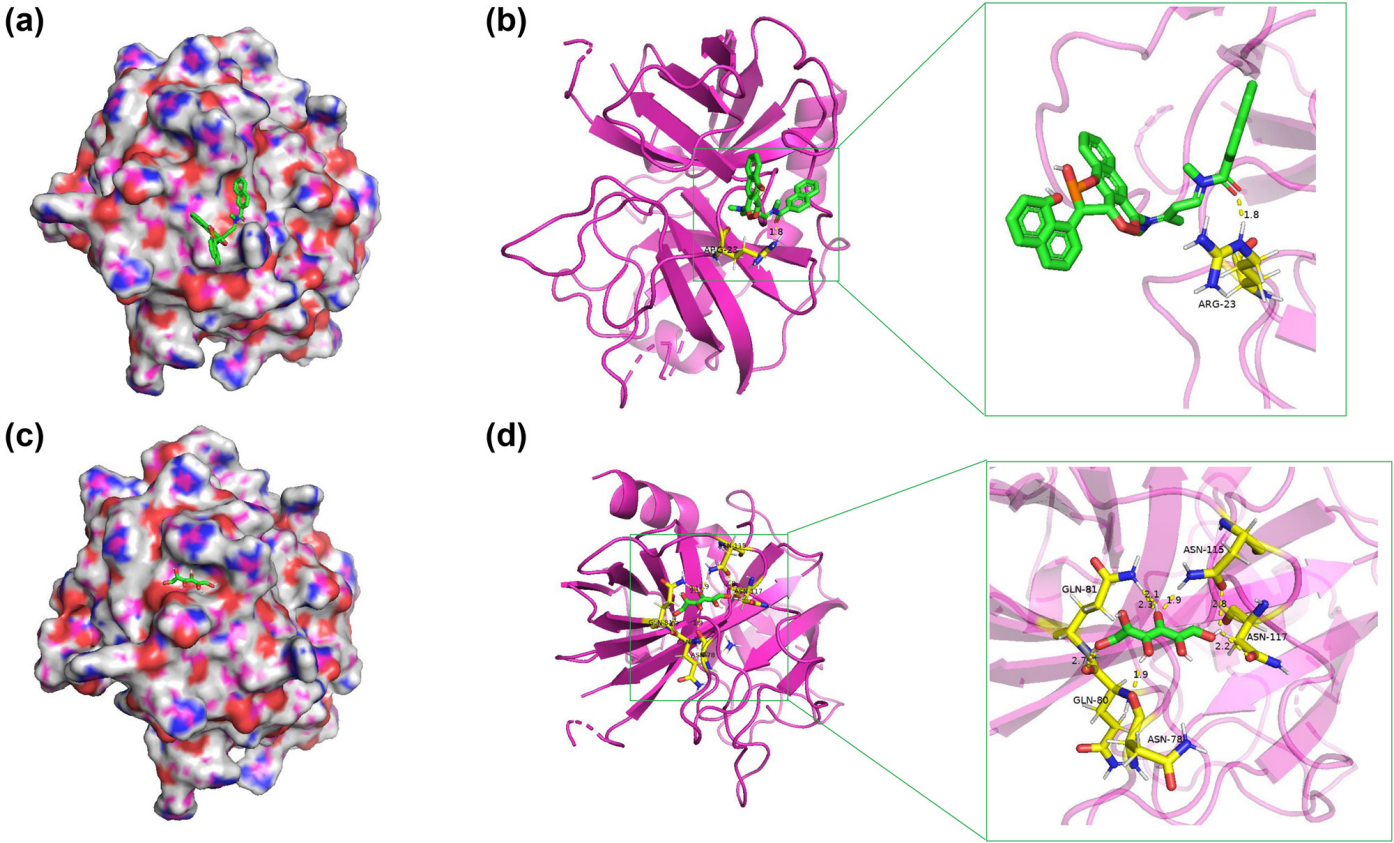
Molecular docking analysis. CTSG protein binding to CH (a and b) and MAN (c and d) was visualized with PyMOL software. A 3D surface structure of the CTSG receptor and the small molecular ligand is presented on the left side of the figure (a and c). The binding pattern of the small molecular ligand to the CTSG receptor is presented on the right side of the figure (b and d).

In the present study, the Dgibd database was applied to screen the potential small molecular compounds for CTSG genes. Our findings showed that CHEMBL374027 (CH) and MANNITOL (MAN) were the targeted drugs of CTSG. Subsequently, the molecular docking analysis was performed to further confirm the above results. Our findings showed that CTSG binds to CH with a binding energy of −4.83 kcal/mol and MAN at −7.05 kcal/mol. As presented in Figure 8, there were interactions and binding modes between the CTSG protein and small molecules (CH and MAN). All results showed that CH and MAN had a good potential to target CTSG genes.

## 4 Discussion

Despite advancements in cancer research and treatment, HNSC still has a bleak outlook with little improvement in prognosis over the last few decades [18]. Thus, the identification of new biomarkers and a deeper understanding of the molecular mechanisms involved in HNSC are essential for the development of more effective diagnostic and treatment approaches. In the present study, three LRGs (CLTCL1, LAPTM4B, and CTSG) were identified for predicting the prognosis of HNSC patients. Among these prognostic genes, only the CTSG gene proved to be an independent prognostic factor for HNSC patients. In addition, our findings indicated that low CTSG expression was associated with poor overall survival, disease-specific survival, and progress-free interval in HNSC patients. CTSG is a serine protease mainly found in azurophilic granules of neutrophils [19]. Few studies have shown that CTSG genes were associated with tumor progression and prognosis. However, a recent study has demonstrated that CTSG was a prognostic biomarker for oral squamous cell carcinoma patients [20]. The aim of this study was to examine the biological function and molecular mechanism of CTSG and to determine its potential as a cancer biomarker.

The tumor microenvironment plays a crucial role in the development and progression of cancer as it contains tumor cells that interact with surrounding cells through the lymphatic systems. This interaction influences tumorigenesis and highlights the importance of studying the tumor microenvironment in cancer research [21]. The progression of cancer is heavily influenced by changes in the tumor microenvironment, which includes non-immune and immune cells as well as the extracellular matrix. These changes can have a significant impact on tumor progression [22,23]. In the present study, we also performed DEG analysis and functional enrichment analysis to assess the potential function of CTSG expression in subgroups of HNSC patients. The GSEA results showed CTSG-associated DEGs were mainly implicated in inflammation- and immune-related pathways, such as neutrophil degranulation, signaling by interleukins, natural killer cell-mediated cytotoxicity, B-cell receptor signaling pathway, inflammatory response pathway, etc. Previous studies have revealed that CTSG could activate matrix metalloproteinases and regulate inflammation, and play an important role in a variety of inflammatory diseases such as acute respiratory distress syndrome, rheumatoid arthritis, chronic obstructive pulmonary disease, and periodontitis [19,24,25]. In addition, CTSG genes were shown to be important for maintaining neutrophil-medicated acute histopathology and fibrosis after renal ischemia/reperfusion injury [26]. CTSG could regulate the level of soluble forms of TNF-α and promote the pro-inflammatory effect of neutrophils [27]. Moreover, immune inflammation played a primary role in drug resistance, clinical outcomes, and tumor microenvironment in HNSC [28]. Specifically, interleukin 2 immunotherapy showed promising results in the treatment of HNSC [29,30].

Another important finding was that CTSG expression was positively correlated with the level of infiltration of immune cells (mast cells, DC, eosinophils, neutrophils, B cells, macrophages, CD4 T cells, and CD8 T cells) in HNSC patients. Our findings imply that CTSG plays a significant role in promoting tumor progression by facilitating communication between tumor cells and their inflammatory microenvironment. Increasing evidence suggests that immune cells and their biomarkers can act as therapeutic targets or prognostic markers or participate in tumor progression. An immune-related gene prognostic index differentiated prognosis and served as a potential biomarker in HNSC patients [31]. A recent study revealed the importance of B cells in prognosis and responses to PD-1 blockade in HPV-associated HNSC patients [32]. CD8 T cells were independent prognostic biomarkers in HNSC patients, and patients with low levels of B cells, CD8 T cells, and endothelial cells had a worse prognosis [33]. The poor prognosis of HNSC patients was associated with the presence of tumor-associated macrophages [34]. Neutrophils increased the invasiveness of oral squamous cell carcinoma by activating invadopodia [35]. All these pieces of evidence further showed that CTSG genes impacted the prognosis of HNSC patients by affecting tumor immune cell infiltration. However, the relationship between immunoregulatory cells and CTSG expression requires further investigation.

In our study, two potential drugs (CH and MAN) with high affinity to CTSG protein were identified. Among them, MAN exerted the nephroprotective effect in HNSC patients with cisplatin therapy [36]. Although these potential compounds should be investigated in depth for specific mechanisms, our findings implied their potential in tumor immunotherapy.

## 5 Conclusion

Our findings suggested that CTSG was an independent prognostic biomarker in HNSC patients and was associated with tumor immune cell infiltration in patients. These results laid a theoretical foundation for further exploration of the role of CTSG in HNSC patients.

## Supplementary Material

Supplementary Figure

## References

[1] Sung H, Ferlay J, Siegel RL, Laversanne M, Soerjomataram I, Jemal A, et al. Global Cancer Statistics 2020: GLOBOCAN estimates of incidence and mortality worldwide for 36 cancers in 185 countries. CA Cancer J Clin. 2021;71(3):209–49. 10.3322/caac.21660 33538338

[2] Johnson DE, Burtness B, Leemans CR, Lui VWY, Bauman JE, Grandis JR. Head and neck squamous cell carcinoma. Nat Rev Dis Primers. 2020;6(1):92. 10.1038/s41572-020-00224-3 PMC7944998 33243986

[3] Svider PF, Blasco MA, Raza SN, Shkoukani M, Sukari A, Yoo GH, et al. Head and Neck Cancer. Otolaryngol–Head Neck Surg Off J Am Acad Otolaryngol–Head Neck Surg. 2017;156(1):10–3. 10.1177/0194599816674672 28045631

[4] Brahmer JR, Drake CG, Wollner I, Powderly JD, Picus J, Sharfman WH, et al. Phase I study of single-agent anti–programmed death-1 (MDX-1106) in refractory solid tumors: Safety, clinical activity, pharmacodynamics, and immunologic correlates. J Clin Oncol. 2023;41(4):715–23. 10.1200/JCO.22.02270 36706735

[5] Russell S, Angell T, Lechner M, Liebertz D, Correa A, Sinha U, et al. Immune cell infiltration patterns and survival in head and neck squamous cell carcinoma. Head Neck Oncol. 2013;5(3):24. PMC3979926 24723971

[6] Leemans CR, Snijders PJF, Brakenhoff RH. The molecular landscape of head and neck cancer. Nat Rev Cancer. 2018;18(5):269–82. 10.1038/nrc.2018.11 29497144

[7] Yang C, Wang X. Lysosome biogenesis: Regulation and functions. The. J Cell Biol. 2021;220(6):e202102001. 10.1083/jcb.202102001 PMC8105738 33950241

[8] Perera RM, Zoncu R. The lysosome as a regulatory hub. Annu Rev Cell Dev Biol. 2016;32:223–53. 10.1146/annurev-cellbio-111315-125125 PMC9345128 27501449

[9] Cisneros J, Belton TB, Shum GC, Molakal CG, Wong YC. Mitochondria-lysosome contact site dynamics and misregulation in neurodegenerative diseases. Trends Neurosci. 2022;45(4):312–22. 10.1016/j.tins.2022.01.005 PMC8930467 35249745

[10] Root J, Merino P, Nuckols A, Johnson M, Kukar T. Lysosome dysfunction as a cause of neurodegenerative diseases: Lessons from frontotemporal dementia and amyotrophic lateral sclerosis. Neurobiol Dis. 2021;154:105360. 10.1016/j.nbd.2021.105360 PMC8113138 33812000

[11] Radisavljevic Z. Lysosome activates AKT inducing cancer and metastasis. J Cell Biochem. 2019;120(8):12123–7. 10.1002/jcb.28752 31021471

[12] Wang S, Ni HM, Chao X, Wang H, Bridges B, Kumer S, et al. Impaired TFEB-mediated lysosomal biogenesis promotes the development of pancreatitis in mice and is associated with human pancreatitis. Autophagy. 2019;15(11):1954–69. 10.1080/15548627.2019.1596486 PMC6844531 30894069

[13] Ge W, Li D, Gao Y, Cao X. The roles of lysosomes in inflammation and autoimmune diseases. Int Rev Immunol. 2015;34(5):415–31. 10.3109/08830185.2014.936587 25075736

[14] Tang T, Yang ZY, Wang D, Yang XY, Wang J, Li L, et al. The role of lysosomes in cancer development and progression. Cell Biosci. 2020;10(1):131. 10.1186/s13578-020-00489-x PMC7677787 33292489

[15] Li T, Fan J, Wang B, Traugh N, Chen Q, Liu JS, et al. TIMER: A web server for comprehensive analysis of tumor-infiltrating immune cells. Cancer Res. 2017;77(21):e108. 10.1158/0008-5472.CAN-17-0307 PMC6042652 29092952

[16] Sun D, Wang J, Han Y, Dong X, Ge J, Zheng R, et al. TISCH: A comprehensive web resource enabling interactive single-cell transcriptome visualization of tumor microenvironment. Nucleic Acids Res. 2021;49(D1):D1420–30. 10.1093/nar/gkaa1020 PMC7778907 33179754

[17] Freshour SL, Kiwala S, Cotto KC, Coffman AC, McMichael JF, Song JJ, et al. Integration of the drug-gene interaction database (DGIdb 4.0) with open crowdsource efforts. Nucleic Acids Res. 2021;49(D1):D1144–51. 10.1093/nar/gkaa1084 PMC7778926 33237278

[18] Bai S, Zhang P, Zhang JC, Shen J, Xiang X, Yan YB, et al. A gene signature associated with prognosis and immune processes in head and neck squamous cell carcinoma. Head Neck. 2019;41(8):2581–90. 10.1002/hed.25731 30839132

[19] Pérez-Is L, Ocaña MG, Montes AH, Carton JA, Álvarez V, Meana Á, et al. The N125S polymorphism in the cathepsin G gene (rs45567233) is associated with susceptibility to osteomyelitis in a Spanish population. PLoS One. 2019;14(10):e0220022. 10.1371/journal.pone.0220022 PMC6812796 31647805

[20] Huang GZ, Wu QQ, Zheng ZN, Shao TR, Li F, Lu XY, et al. Bioinformatics analyses indicate that Cathepsin G (CTSG) is a potential immune-related biomarker in oral squamous cell carcinoma (OSCC). OncoTargets Ther. 2021;14:1275–89. 10.2147/OTT.S293148 PMC7920606 33658795

[21] Arneth B. Tumor microenvironment. Medicina (Kaunas, Lithuania). 2019;56(1):15. 10.3390/medicina56010015 PMC7023392 31906017

[22] Swartz MA, Iida N, Roberts EW, Sangaletti S, Wong MH, Yull FE, et al. Tumor microenvironment complexity: Emerging roles in cancer therapy. Cancer Res. 2012;72(10):2473–80. 10.1158/0008-5472.CAN-12-0122 PMC3653596 22414581

[23] Junttila MR, de Sauvage FJ. Influence of tumour micro-environment heterogeneity on therapeutic response. Nature. 2013;501(7467):346–54. 10.1038/nature12626 24048067

[24] Korkmaz B, Moreau T, Gauthier F. Neutrophil elastase, proteinase 3 and cathepsin G: physicochemical properties, activity and physiopathological functions. Biochimie. 2008;90(2):227–42. 10.1016/j.biochi.2007.10.009 18021746

[25] Korkmaz B, Horwitz MS, Jenne DE, Gauthier F. Neutrophil elastase, proteinase 3, and cathepsin G as therapeutic targets in human diseases. Pharmacol Rev. 2010;62(4):726–59. 10.1124/pr.110.002733 PMC2993259 21079042

[26] Shimoda N, Fukazawa N, Nonomura K, Fairchild RL. Cathepsin g is required for sustained inflammation and tissue injury after reperfusion of ischemic kidneys. Am J Pathol. 2007;170(3):930–40. 10.2353/ajpath.2007.060486 PMC1864870 17322378

[27] Mezyk-Kopeć R, Bzowska M, Bzowska M, Mickowska B, Mak P, Potempa J, et al. Effects of elastase and cathepsin G on the levels of membrane and soluble TNFalpha. Biol Chem. 2005;386(8):801–11. 10.1515/BC.2005.094 16201876

[28] Zhu L, Wang Y, Yuan X, Ma Y, Zhang T, Zhou F, et al. Effects of immune inflammation in head and neck squamous cell carcinoma: Tumor microenvironment, drug resistance, and clinical outcomes. Front Genet. 2022;13:1085700. 10.3389/fgene.2022.1085700 PMC9790931 36579330

[29] O’Malley BW Jr, Sewell DA, Li D, Kosai K, Chen SH, Woo SL, et al. The role of interleukin-2 in combination adenovirus gene therapy for head and neck cancer. Mol Endocrinol (Baltimore, MD). 1997;11(6):667–73. 10.1210/mend.11.6.0012 9171230

[30] O’Malley BW Jr, Li D, McQuone SJ, Ralston R. Combination nonviral interleukin-2 gene immunotherapy for head and neck cancer: From bench top to bedside. Laryngoscope. 2005;115(3):391–404. 10.1097/00005537-200503000-00002 15744147

[31] Chen Y, Li ZY, Zhou GQ, Sun Y. An immune-related gene prognostic index for head and neck squamous cell carcinoma. Clin Cancer Res Off J Am Assoc Cancer Res. 2021;27(1):330–41. 10.1158/1078-0432.CCR-20-2166 33097495

[32] Kim SS, Shen S, Miyauchi S, Sanders PD, Franiak-Pietryga I, Mell L, et al. B cells improve overall survival in HPV-associated squamous cell carcinomas and are activated by radiation and PD-1 blockade. Clin Cancer Res Off J Am Assoc Cancer Res. 2020;26(13):3345–59. 10.1158/1078-0432.CCR-19-3211 PMC7334097 32193227

[33] Zhang Y, Li L, Zheng W, Zhang L, Yao N. CD8(+) T-cell exhaustion in the tumor microenvironment of head and neck squamous cell carcinoma determines poor prognosis. Ann Transl Med. 2022;10(6):273. 10.21037/atm-22-867 PMC9011205 35434003

[34] Li B, Ren M, Zhou X, Han Q, Cheng L. Targeting tumor-associated macrophages in head and neck squamous cell carcinoma. Oral Oncol. 2020;106:104723. 10.1016/j.oraloncology.2020.104723 32315971

[35] Glogauer JE, Sun CX, Bradley G, Magalhaes MA. Neutrophils increase oral squamous cell carcinoma invasion through an invadopodia-dependent pathway. Cancer Immunol Res. 2015;3(11):1218–26. 10.1158/2326-6066.CIR-15-0017 26112922

[36] Hägerström E, Lindberg L, Bentzen J, Brødbæk K, Zerahn B, Kristensen B. The nephroprotective effect of mannitol in head and neck cancer patients receiving cisplatin therapy. Clin Med Insights Oncol. 2019;13:1179554918821320. 10.1177/1179554918821320 PMC6327333 30670924

