# Supplementary material for: Identification of lysosome-related genes in connection with prognosis and immune cell infiltration for drug candidates in head and neck cancer
Source: Open Life Sci. 2023 Aug 9;18(1):20220660. doi: 10.1515/biol-2022-0660 (PMC10426727; doi:10.1515/biol-2022-0660)
Supplement: Supplementary Figure [file biol-2022-0660-sm.pdf]

Supplementary material

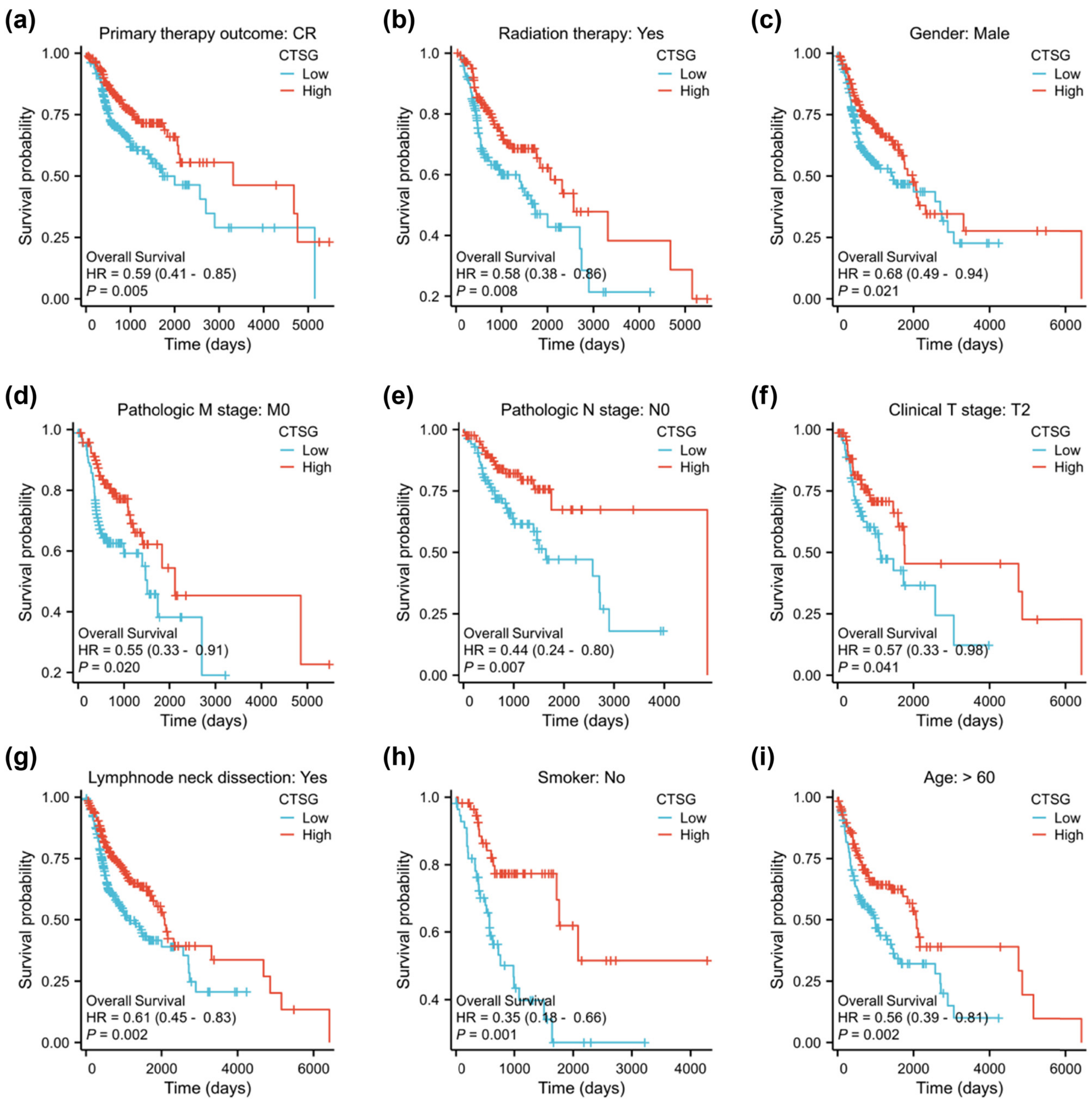

Figure S1: Assessment of CTSG's prognostic value in HNSC. The overall survival for CR therapy outcome (a), radiation therapy (b), male (c), M0 stage (d), N0 stage (e), T2 stage (f), lymphnode neck dissection (g), smoker (h), and age above 60 (i).

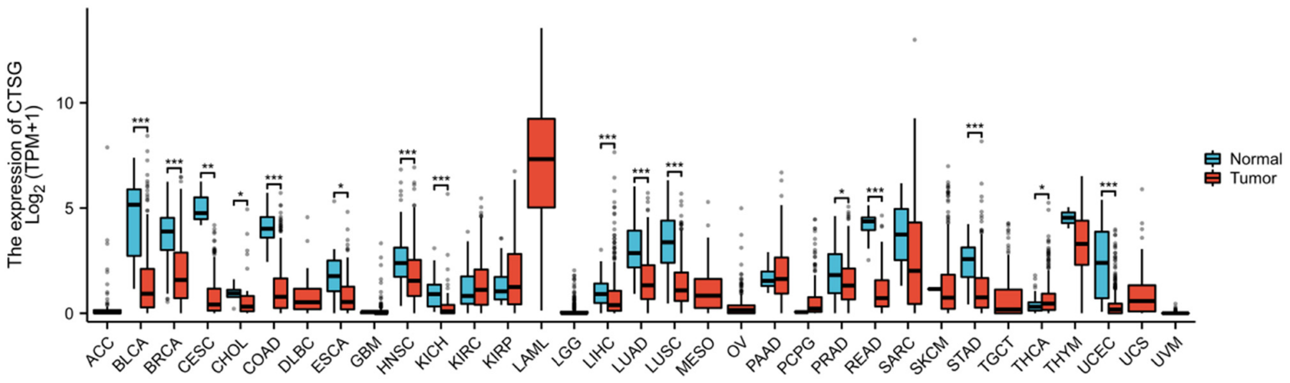

Figure S2: Pan-cancer analysis of the expression of CTSG. *$p < 0.05$, **$p < 0.01$, ***$p < 0.001$.

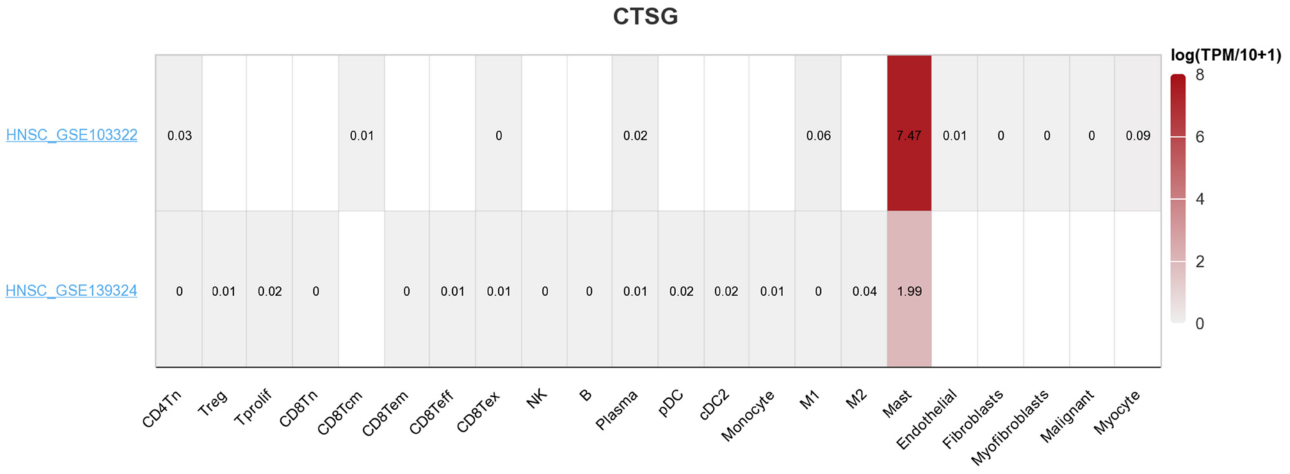

Figure S3: CTSG gene was expressed in tumor microenvironment-related cells.
